# Effect of Gender, Disease Duration and Treatment on Muscle Strength in Myasthenia Gravis

**DOI:** 10.1371/journal.pone.0164092

**Published:** 2016-10-14

**Authors:** Gülsenay Citirak, Sanja Cejvanovic, Henning Andersen, John Vissing

**Affiliations:** 1 Neuromuscular Research Unit, Department of Neurology, Rigshospitalet, University of Copenhagen, Copenhagen, Denmark; 2 Department of Neurology, Aarhus University Hospital, Aarhus, Denmark; University of California, Davis, UNITED STATES

## Abstract

**Introduction:**

The aim of this observational, cross-sectional study was to quantify the potential presence of muscle weakness among patients with generalized myasthenia gravis (gMG). The influence of gender, treatment intensity and disease duration on muscle strength and disease progression was also assessed.

**Methods:**

Muscle strength was tested in 8 muscle groups by manual muscle testing and by hand-held dynamometry in 107 patients with gMG and 89 healthy age- and gender-matched controls. Disease duration, severity and treatment history were reviewed and compared with muscle strength.

**Results:**

Patients had reduced strength in all tested muscle group compared to control subjects (p<0.05). Women with gMG were stronger than men (decrease in strength 22.6% vs. 32.7% in men, P<0.05). Regional differences in muscle weakness were also evident, with proximal muscles being more affected. Interestingly, muscle strength did not correlate with disease duration and treatment intensity.

**Conclusions:**

The results of this study show that in patients with gMG; 1) there is significant muscle weakness, 2) muscle weakness is more pronounced in men than women, 3) shoulder abductors, hip flexors, and neck muscles are the most affected muscle groups and 4) disease duration or treatment intensity alone are not predictors of loss of muscle strength in gMG.

## Introduction

Myasthenia Gravis (MG) is an autoimmune neuromuscular disorder characterized by fluctuating strength of voluntary muscles. The disease is highly heterogeneous with respect to age of onset, pattern of muscular involvement, severity and clinical course [1;2]. The majority of patients with MG present with involvement of ocular muscles. Frequently, muscle fatigability progresses during the first years of the disease to involve bulbar and limb muscles (generalized MG (gMG) [3–5]).

In all non-hereditary forms of myasthenia, the underlying pathophysiology is ascribed to circulating autoantibodies directed against elements of the synapse in the neuromuscular junction. In approximately 80% of gMG patients, autoantibodies can be measured, which are targeted at the acetylcholine receptor (AChR) [6–10]. This immunological attack can, with time, result in a less folded postsynaptic membrane at the neuromuscular junction (NMJ), and a reduction in the number of functional AChRs. This results in impaired neuromuscular transmission, and subsequent muscle weakness and fatigability [7;9].

Current treatment options include acetylcholinesterase inhibitors, which are effective in enhancing stimulation of functionally competent AChRs. However, acetylcholinesterase inhibitors do not prevent the underlying autoimmune process, and therefore do not prevent the destruction of functional AChRs, which immunomodulators are capable of to some extent [4;10;11].

The pattern of weakness in most gMG patients is well established and recognized [4]. However, knowledge regarding muscle force in general, and the degree of reduced muscle force among MG patients is limited. No large studies on muscle strength in patients with gMG have been conducted. In the present study, we examined the muscle strength by dynamometry in a large cohort of patients with gMG. We also assessed whether weakness, if present, was related to gender, treatment intensity, disease severity and duration or presented with a particular pattern of involvement. Therefore, the main purpose was to assess static muscle strength and not endurance, which most often is the focus of MG investigations, to explore whether continued immunological attack on the NMJ results in loss of maximal muscle force.

Our hypotheses were 1) that patients with gMG have decreased muscle strength compared to healthy age- and gender-matched controls, and 2) that the degree of decreased muscle strength could relate to gender, particular muscle groups, disease duration or current or past treatment intensity.

## Methods

### Study design

This is an observational, cross-sectional cohort study.

### Subjects

**Fig 1:**
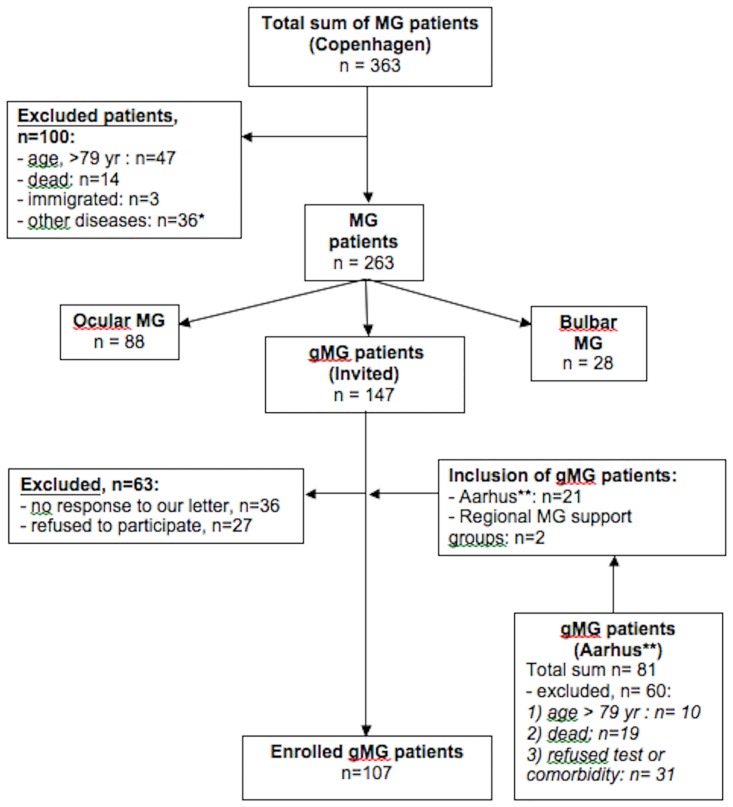
Flow chart of enrolled patients with generalized myasthenia gravis in the study. *Other diseases in the excluded patients included: Ehlers-Danlos syndrome (1 patient), severe osteoarthritis (6 patients), severe psychiatric diseases (1 patient), cancer (9 patients), other neurological diseases (19 patients). ** Patients examined in Aarhus, were primarily from outside Aarhus County.

Between June 2009 and June 2013, a total of 107 patients diagnosed with gMG were enrolled in the study from the Neuromuscular Clinic at the National Hospital in Copenhagen (84 patients), Department of Neurology at the University Hospital in Aarhus (21 patients) and from regional support groups (2 patients). Thirty-eight patients, in whom we have previously reported muscle strength [12], also participated in this study. All patients, 18–79 years old, had a confirmed diagnosis of gMG. They all had a typical clinical presentation for gMG and a clear treatment response to acetylcholine esterase inhibitors. Ninety-three percent carried acetylcholine receptor antibodies in serum, and in the remaining 7%, with no antibodies, neurophysiological investigations demonstrated increased jitter or a decrement to repetitive stimulation in all. Exclusion criteria were the presence of other disorders that would impair muscle strength or promote fatigue. Patients were included in the study according to the flowchart shown in Fig 1.

A group of 89 gender- and age-matched healthy subjects were recruited from the hospital staff, and relatives and friends of the patients. In addition to reporting no history of MG, these healthy subjects were validated according to the same exclusion criteria as the patients. Muscle strength in this healthy group of volunteers acted as reference for normal strength in the patients, sub-grouped according to gender and age.

All participants volunteered to participate and an informed written consent was obtained for each prior to their inclusion in the study. The Danish National Committee on Biomedical Research Ethics of Copenhagen approved the study (approval # H-4-2010-113).

### Clinical examination

Clinical profiles and history of treatments from disease onset to the test date were reviewed from medical records and patient interviews. The disease severity was evaluated according to the Myasthenia Gravis Foundation of America (MGFA) clinical classification [13] (grade 0, no symptoms; grade I, ocular muscle weakness only; grade II, mild generalized weakness, grade III, moderate generalized weakness; grade IV, severe generalized weakness; grade V, intubation required). No changes were made in the patients’ treatment regime prior to participation, and all were tested within 3 hours after last dose of pyridostigmine. The patients were on a stable pyridostigmine dose for at least 1 month before assessment. The rationale for standardizing the timing of strength testing relative to dosing of pyridostigmine was to make sure that patients had comparable treatment effects from pyridostigmine, in the time-frame of effectiveness of the drug, so that it reflects the daily clinical status of the patients.

### Muscle strength testing

#### Manual muscle testing

**Fig 2:**
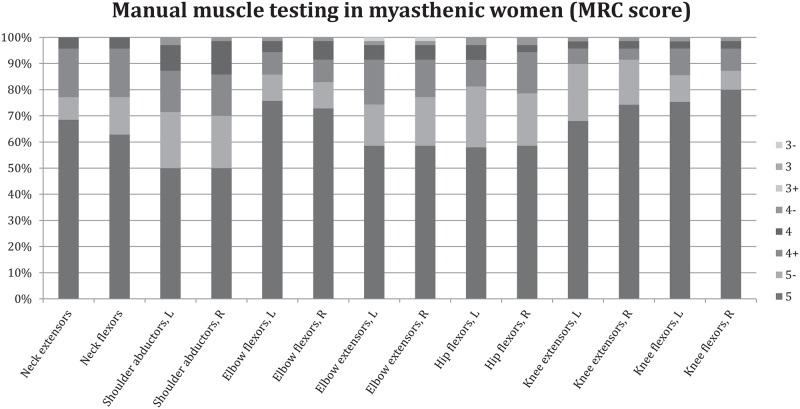
Manual muscle testing in eight muscle groups using the Medical Research Council (MRC) score in female patients with generalized myasthenia gravis. L = left side & R = right side.

**Fig 3:**
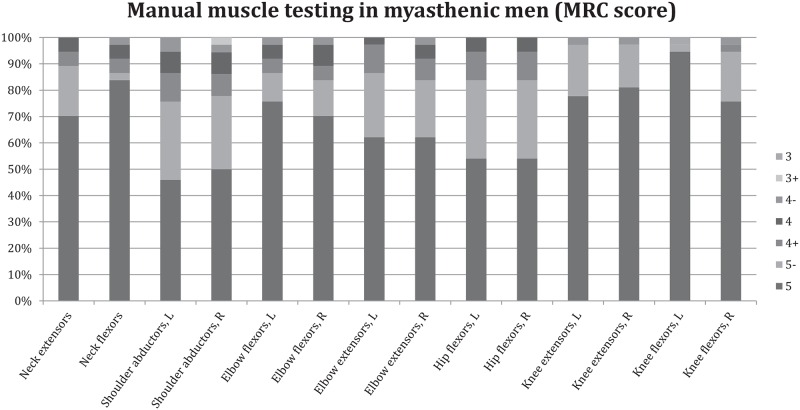
Manual muscle testing in eight muscle groups using the Medical Research Council (MRC) score in male patients with generalized myasthenia gravis. L = left side & R = right side.

Prior to testing with dynamometry, muscle strength was evaluated by manual muscle testing (MMT) of eight muscle groups (see Figs 2 and 3 for included muscle groups) by using the modified Medical Research Council (MRC) score where the grades 3, 4 and 5 are subdivided [14].

#### Assessment of muscle strength by dynamometry

The isometric muscle strength of the same eight muscle groups was measured using a CITEC hand-held dynamometer type CT3002, which has a muscle force measuring capacity of up to 500 Newton. The reliability of using hand-held dynamometers to measure muscle force has been confirmed several times [15–18], and has successfully been used to measure muscle strength in neuromuscular diseases in general [19], and specific muscular diseases such as spinal muscular atrophy [15], chronic inflammatory demyelinating polyneuropathy [20], Duchenne and limb-girdle muscular dystrophies [21], and facioscapulohumeral dystrophy [22], but not gMG specifically. Two examiners (GC, 121 subjects and SC, 75 subjects) performed all strength tests. The two examiners trained together and adopted the same muscle dynamometry technique, so that test results were comparable. Inter-observer variance was found to be below 10%. The subjects were instructed to perform a maximal voluntary isometric contraction (MVIC) for each muscle group. Three consecutive attempts, separated by 15–30 s, were made for each strength measure. The body position for measuring the strength was adjusted to the following protocol: neck flexion and extension were measured in sitting position with a firm back and head up at 90° from horizontal; shoulder abduction was measured in sitting position with the shoulder abducted 90°, elbow flexed 135°and forearm pronated with no fixation; elbow flexion and extension were determined in the supine position, with shoulder adducted, elbow flexed 90°and forearm supinated with no fixation. Hip flexion was determined in the supine position with hip and knee flexed 90°. Knee flexion and extension were measured in prone position with knee flexed 90°. All the muscle groups, except the neck, were tested bilaterally in all subjects.

Strength testing lasted one hour on average for each subject. No adverse events were noted at any time, and none of the participants reported any signs of muscle injury following the tests. Twenty-one subjects experienced muscle cramps in hamstrings on testing of the strength in knee flexion, but any pain from this subsided within a few minutes.

### Statistical analyses

Data collected during patient interviews and from the medical records are reported descriptively, and expressed as mean values with standard deviation and range and as percentage of normal. Potential differences among groups were evaluated by Student’s unpaired t-test and analysis of variance (ANOVA) when appropriate.

The test with the highest force obtained during the three trials for each muscle group was used for data analysis. Analysis of a potential correlation between muscle strength and treatment intensity was examined for each muscle group.

Treatment intensity was evaluated as described in the following. A score for treatment intensity was assigned for total disease duration and another score was assigned for treatment intensity during the six months before the test date. Treatments were assigned a score according to the intensity of the treatment as follows: pyridostigmine (mestinon) = 1, corticosteroids (prednisone) or a mild immunosuppressive agent (azathioprine, mycophenolate mofetil or methrotrexate) = 2, combination of corticosteroids and a mild immunosuppressive agent or the combination of two mild immunosuppressive agents = 3, thymectomy = 1, other immunosuppressants or immunomodulators (cyclosporine, cyclophosphamide, tacrolimus, rituximab, plasmapheresis and intravenous immunglobulins (IVig)), when they were used because of insufficient response to prednisone or the milder immunosuppressant mentioned above = 4.

Analysis was performed using the SAS software package, v. 9.2 for Windows (SAS Institute, Cary, N.C., USA). Statistical significance was set at P<0.05.

## Results

### Basic characteristics of participants

The demographic and clinical profiles of the 107 patients enrolled in the study are shown in Table 1. The baseline characteristics show an overall similarity between the male and female groups, except for age at study entry. There was an over representation of female patients in the study with a female/male ratio of 1.9. The age of healthy controls was 49±15 (22–73) for women (n = 50) and 48±17 (21–79) for men (n = 39).

**Table 1:** Clinical profile and treatment of 107 patients with generalized myasthenia gravis.

**Demographic information**	
Number of patients studied	107
Gender (M/F)	37/70
Age, mean±SD (range)	
• Female	52±16 (21–79)
• Male	61±16 (23–79)
MG based on diagnostic methods:	
• Electrophysiologic testing (M/F)	8* (2/6)
• Acetylcholine receptor antibody positive (M/F)	99† (35/64)
Years since MG diagnosis, (range)	12.7±11.2 (0.5–60.6)
• Female	12.6±10.3 (0.5–55.6)
• Male	12.9±13.0 (1.0–60.6)
Years since MG onset, (range)‡	14.2±11.4 (1.5–61.6)
• Female	14.2±10.4 (1.5–56.3)
• Male	14.3±13.3 (2.3–61.6)
Years from disease onset to diagnosis, (range)‡	1.6±2.9 (0–25.6)
• Female	2.0±3.5 (0–25.6)
• Male	1.1±1.0 (0–4.7)
MGFA classification	
• Grade II (M/F)	64 (25/39)
• Grade III (M/F)	29 (9/20)
• Grade IV (M/F)	14 (3/11)
**Therapeutic parameters**	
Medications; past therapy (M/F) / current therapy (M/F)	
• Pyridostigmine	107 (37/70) / 84 (28/56)
• Prednisone	84 (34/50) / 25 (10/15)
• Azathioprine	73 (29/44) / 49 (20/29)
• Mycophenolate Mofetil	11 (3/8) / 4 (1/3)
• Methotrexate	6 (2/4) / 5 (2/3)
• Cyclosporine	7 (1/6) / 2 (0/2)
• Cyclophosphamide	4 (1/3) / 1 (0/1)
• Plasmapheresis	23 (10/13) / 2 (1/1)
• IVig	36 (13/23) / 9 (3/6)
• Tacrolimus	1 (0/1) / 1 (1/0)
• Rituximab	2 (0/2) / 0
Thymectomy	49 (12/37)
MG treatment score (last 6 mo.), mean±SD (range)	3.1±1.8 (0–10)
• Female	3.1±1.7 (0–8)
• Male	3.3±1.9 (1–10)
MG treatment score (all yrs.), mean±SD (range)	2.8±1.2 (0.9–6.9)
• Female	2.69±1.23 (0.9–6.9)
• Male	2.97±1.02 (1.0–5.1)

*Patients who were diagnosed by electrophysiological findings were all negative for acetylcholine receptor antibodies.

^†^Most of the patients had also undergone an electrophysiological testing.

^‡^The values are based on 101 patients due to missing information about exact MG onset in 6 patients (3 men and 3 women). Unless otherwise stated, values are mean±SD.

mo. = months, yrs. = years.

The disease duration from time of diagnosis was similar in women (12.6±10.2 years) and men (12.9±13.0 years). The time from symptom onset to a verified diagnosis of MG was not significantly different between men (1.1±1.0 years) and women (2.0±3.5 years). Percentage of patients who were diagnosed with gMG before the age of 50 was 59.8%, and was higher in women (68.6%) than men (43.2%, p<0.0001 (95%CI 0.1–0.4)). In contrast, men out-numbered women in persons diagnosed with gMG after age 50 years (women: 31.4% and men: 56.8%, p<0.0001 (95%CI 0.3–0.6)).

The majority of the gMG group (60%) had mild disease according to MGFA. Only 13% scored grade IV on the MGFA scale.

### Manual muscle testing

Weakness was defined as an MRC score of 5- or less. Both female and male groups scored a median MRC grade of 5 in all muscle groups with the exception of shoulder abductors, which were graded a median MRC grade of 5-.

However, for each muscle group, there were patients, both in the female and male groups, who had muscle weakness of MRC grade 5-, 4 and 3 in some groups. The percentage of patients who had at least one grade under 5 was 33% in both men and women. Figs 2 and 3 illustrate the results for the manual muscle testing for gMG female and male patients, separately.

### Isometric muscle strength—dynamometry

**Fig 4:**
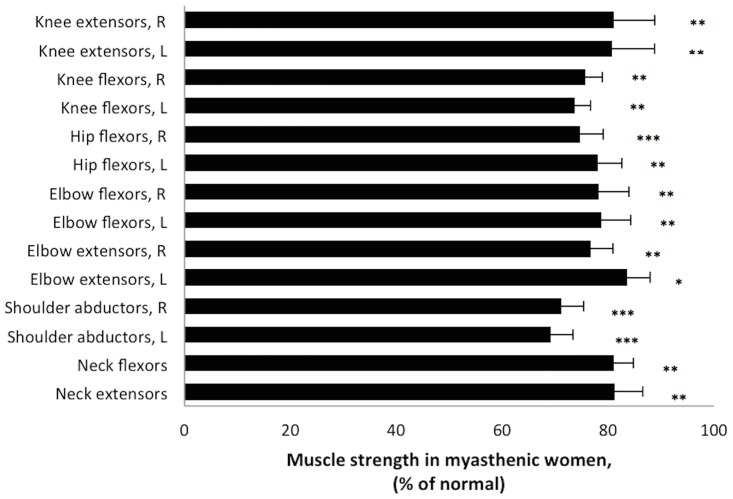
Muscle strength in women with generalized myasthenia gravis. Muscle strength in eight muscle groups in myasthenic women assessed by hand-held dynamometry, expressed as percentage of mean normative values (corresponding to 100%). L = left side and R = right side. * = p<0.05, ** = p<0.005 and *** = p<0.0001.

**Fig 5:**
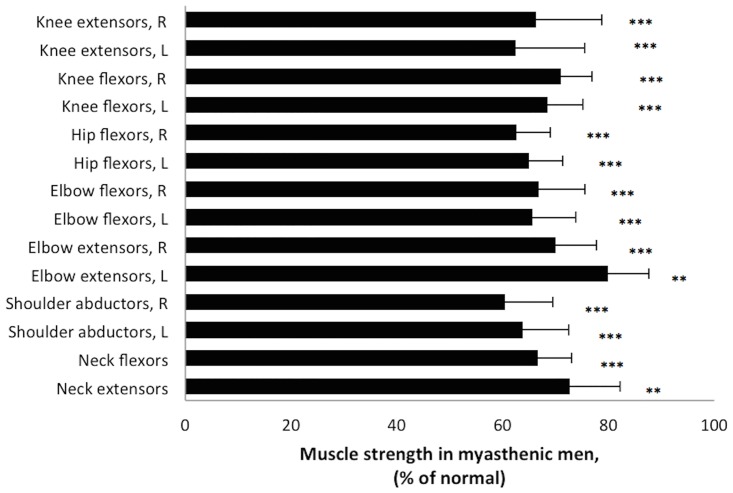
Muscle strength in men with generalized myasthenia gravis. Muscle strength in eight muscle groups in myasthenic men assessed by hand-held dynamometry, expressed as percentage of mean normative values (corresponding to 100%). L = left side and R = right side. ** = p<0.005 and *** = p<0.0001.

Figs 4 and 5 illustrate the muscle strength in the 107 patients, expressed as the percentage of normal strength found in the age- and gender-matched controls. Men with gMG were on average 13 years older than the male control group. However, when analyzing the strength in strictly age-matched controls (n = 27, mean age 57.6±12.2, age range (37–79)) vs. the patients, the results were the same as when comparing the whole control group (n = 39). There was only a 4% difference in strength between the age-matched group of controls and those who were younger, and we have therefore used the pooled results from all controls to compare muscle strength with.

Overall, difference in muscle strength reached significance between patients and controls in all eight muscle groups. On repeated testing, strength measures in individual patients did not vary, indicating that the muscle force reported was not fatigue.

Male controls were significantly stronger than the female controls in all 8 tested muscle groups (p<0.05).

#### Influence of age and gender on muscle strength

**Fig 6:**
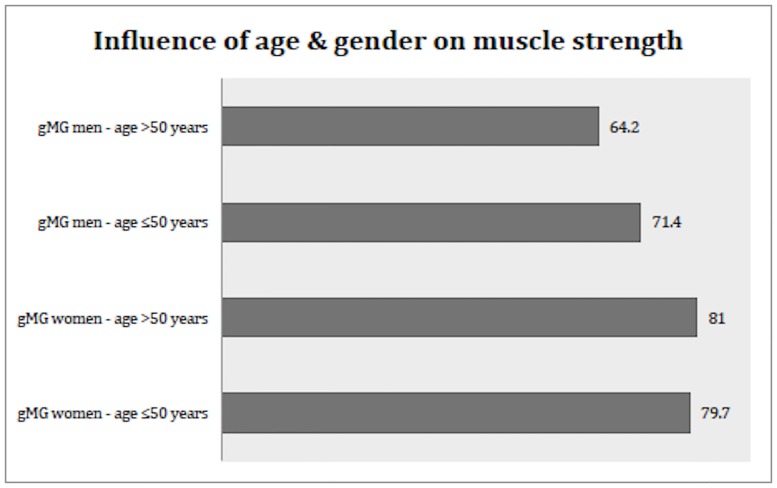
Influence of age and gender on muscle strength in generalized myasthenia gravis. Total composite strength in mysthenic male and female patients under or older than age 50 years, expressed as percentage of normal strength in healthy age- and gender-matched subjects.

Overall, total composite strength was significantly lower in men (67.3% of normal) compared to women with gMG (77.4%), p<0.0001 (95%CI 6.6–13.7). When both age and gender were examined in relation to muscle force, there was no difference in total composite strength between female patients under or older than 50 years. Muscle weakness was more pronounced in men who were 50 years or older (64.2.4% of normal) vs. younger men with gMG (71.4%) (p = 0.002, (95%CI 2.8–11.6)) and women older than 50 years (81%) (p<0.0001, (95% CI 12.4–21.2)) (Fig 6).

#### Topography

In general, muscle weakness was present in all eight muscle groups in both male and female patients regardless of age. However, proximal muscle groups were significantly more affected than distal groups.

The distribution of weakness was generally symmetric in appearance in the majority of patients, however, a marked difference in strength (30 N or more) was found between right and left in shoulder abductors, elbow and knee extensors in about 20 patients.

#### Disease duration and treatment intensity

Table 2 depicts muscle strength in the women and men with gMG, stratified for disease duration.

**Table 2:** Muscle strength according to disease duration in men and women with generalized myasthenia gravis.

**Disease Duration—Women**	**≤5 years, (n = 15)**	**5≤10 years, (n = 22)**	**>10 years, (n = 33)**
Neck extensors	87	77	82
Neck flexors	86	74	84
Shoulder abductors (L/R)	71/73	62/69	73/72
Elbow extensors (L/R)	86/81	81/77	83/75
Elbow flexors (L/R)	82/85	71/71	83/80
Hip flexors (L/R)	85/80	69/68	81/77
Knee extensors (L/R)	89/82	70/76	84/84
Knee flexors (L/R)	75/77	70/76	76/75
**Disease Duration—Men**	**≤5 years, (n = 12)**	**5≤10 years, (n = 10)**	**>10 years, (n = 15)**
Neck extensors	78	73	69
Neck flexors	72	67	62
Shoulder abductors (L/R)	70/73	68/63	57/49
Elbow extensors (L/R)	84/77	82/71	75/64
Elbow flexors (L/R)	69/73	60/62	68/66
Hip flexors (L/R)	64/64	68/66	64/60
Knee extensors (L/R)	59/63	65/70	64/67
Knee flexors (L/R)	62/75	64/60	78/76

Numbers for muscle strength are the percentage of normal strength in healthy gender-matched subjects. L = left side and R = right side.

The aggravation in strength reduction according to disease duration did not reach significance in men or women with gMG. Also, as indicated by this cross-sectional assessment, there did not appear to be a significant worsening of muscle force with age, regardless of gender (p>0.05). However, the shoulder abductors in male patients seemed to have a tendency to decline in strength with disease duration (p = 0.059).

**Fig 7:**
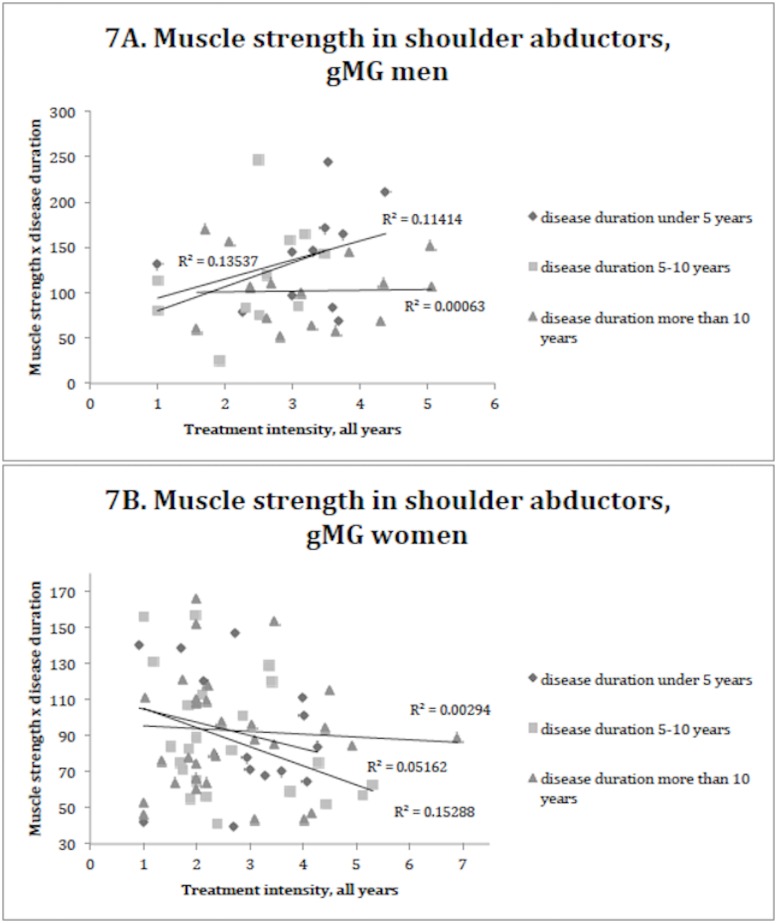
Muscle strength in shoulder abductor muscle groups in gMG men (7A) and women (7B), classified into disease duration <5 years, 5–10 years and >10 years against treatment intensity.

No correlation was found between muscle strength and treatment intensity. When comparing treatment intensity and muscle strength, the treatment intensity was expressed either as 1) the treatment intensity score for the last 6 months before test date, or 2) the treatment intensity score for all the years since diagnosis of MG. Treatment intensity at any time did not show any coherence with muscle strength of the patients. Fig 7 illustrates the lack of correlation between muscle strength and disease duration against treatment intensity in selected proximal muscle groups in female and male patients with gMG.

## Discussion

The main findings of this study are that: 1) patients with gMG have a significant, generalized muscle weakness, 2) men are weaker than women with gMG, 3) weakness is most pronounced in proximal muscle groups, and 4) disease duration or treatment intensity alone are not predictors of loss of muscle strength in gMG.

Two small-scale studies have previously measured force to evaluate weakness in MG [12;23]. Symonette et al. quantified strength and fatigue exclusively in the shoulder muscles of 20 patients with gMG and concluded that muscle strength and fatigue in this muscle group were worse than in healthy controls [23]. In a previous study by us [12], we examined the strength in 38 gMG patients. Due to the small patient sample, we were not able to analyze the relation of strength to medical treatment intensity and disease duration in detail. However, the study also found that patients with gMG have a preferential proximal muscle weakness, and that men are more affected than women, findings that have been significantly strengthened by the findings in the present large cohort of gMG patients.

The cause of static muscle weakness in gMG is not fully understood. What is known, is that the junctional folding is destroyed in auto-immune MG, especially in undertreated MG patients, which restricts the membrane surface available for replacing new AChRs [24]. This in itself will decrease muscle strength, and not just endurance. On top of this known phenomenon, intracellular events related to the excitation-contraction coupling could also be perturbed, but experimental evidence for this is lacking.

The present study demonstrates that dynamometry is much more sensitive in demonstrating mild muscle weakness in patients with gMG compared with manual muscle testing. Therefore, dynamometry seems to be more suitable to monitor the disease condition both in clinical trials and in clinics. Our findings also reveal the topographic differences in muscle strength in women and men with gMG, showing that patients have a more pronounced weakness in proximal upper and lower limb muscles (shoulder abductors and hip flexors) (Figs 4 and 5). This is consistent with many gMG patients having problems climbing stairs and work with their arms above their head. Consistent with a frequent occurrence of head-drop in gMG [4;5;9;25], weakness of neck muscles was also demonstrated to be significant in our study.

Another interesting finding in this study is that muscle strength is more severely affected in men vs. women in most muscle groups. Long-term corticosteroid treatment could have a role and induce a steroid myopathy in some patients. However, this should likely affect women more than men as disease duration is longer in women, and that was not the case in this present study. Since the mean age of the male patients in this study was 61 years and that 57% of them had their gMG onset at age 50 or after, which is a well-known sex difference in onset for gMG [5;25;26], the sex difference in muscle weakness might be explained by a different immunereaction in elderly men vs young women. Another explanation that can support the finding is that a more rapid progression of weakness is detected in the first months of disease onset in men vs. women [5]. Our study demonstrates a difference in severity of muscle weakness among male patients under age 50 vs. age 50 or older, but such a difference could not be detected in women with gMG.

Dynamometer scores are influenced by the examiner as well as the experimental set-up. The force of the examiners was inadequate when testing knee extensors in 5 male controls in the age group 20–59 years, 3 female and 5 male patients in age group 20–59. Thus, values were truncated at a ceiling value of 400 N. Cramps in hamstring muscles were provoked by testing strength in knee flexion in 15 patients and in 8 controls. Consequently, three repeated trials for knee flexion were not always performed in these subjects. Pain from the muscle cramps always subsided within a few minutes.

Our study found no correlation between muscle strength and treatment intensity in the cohort of patients with gMG. There may be several explanations for this. The phenotype of myasthenia is governed by multiple factors, and treatment intensity is just one of them [2;5]. Perhaps most importantly, the natural course of a MG phenotype is highly variable and impacts significantly on the MG clinical course and prognosis [25]. Thus, some patients have severe MG at disease onset and will often have a worse muscle function early on than some patients with longer disease duration. Another reason is that some patients do not respond well to conventional MG treatments, which may indicate the possibility of exacerbating factors or other processes unrelated to gMG that produce weakness [9]. It is most likely that no gMG patients are prescribed immunosuppressive agents if it is not needed. One could therefore speculate that patients on immunosuppressants likely have a more severe disease presentation. But the successful effect of the drugs may counteract the possibility to detect this worse disease severity. Thus, it is not surprising that muscle strength is not linked to treatment intensity. Finally, the fact that patients were not put on treatment in a randomized fashion and that this study is retrospective, could also explain the inability to link disease duration and treatment intensity to current muscle strength. Our study illustrates how variable in phenotype MG presents itself. The severity and age at which the disease presents and gender difference make it difficult to draw conclusions between disease duration and muscle weakness. One can therefore argue that measuring muscle strength is not a predictive marker of disease duration or treatment intensity, but could likely be valuable for follow-up of patients, in a prospective manner.

An association between disease duration and muscle weakness was also absent in our study, which probably relates to the same argument for not detecting an association between treatment intensity and strength as described above. The findings, although cross-sectional in design, seem to suggest that gMG patients are generally well treated from disease onset, which potentially preserves muscle strength during the course of the disease. Future prospective studies should investigate the time-course changes in patients with gMG.
